# Atmospheric bulk depositions: state-of-the-art and European legislative framework with focus on Italy

**DOI:** 10.1007/s11356-024-34338-y

**Published:** 2024-07-23

**Authors:** Giuseppe Ianiri, Gaetano Settimo, Pasquale Avino

**Affiliations:** 1 Department of Environment and Health, Italian National Institute of Health, Viale Regina Elena, 299, 00161 Rome, Italy; 2 Department of Agricultural, Environmental and Food Sciences, University of Molise, Via F. De Sanctis, 86100 Campobasso, Italy; 3 Institute of Atmospheric Pollution Research (IIA), National Research Council (CNR), Rome Research Area-Montelibretti, 00015 Monterotondo Scalo, Italy

**Keywords:** Atmospheric bulk depositions, Legislation, Europe, Persistent organic pollutants, Metals, Public health

## Abstract

The determination of total atmospheric deposition (bulk) is an essential tool to assess the state of environmental contamination and the consequent exposure of the population to persistent organic pollutants (POPs) through the intake of contaminated food. Over the past 20 years, international authorities and the European Union through various pieces of legislation have emphasised the importance of conducting monitoring and studies on depositions to better understand their impact on the environment and human health without setting reference values. Despite the absence of such values, several European countries, through national laws, have adopted limit values and/or guideline values for the deposition fluxes of some organic (dioxins, furans, polychlorinated biphenyls and polycyclic aromatic hydrocarbons) and inorganic persistent pollutants (metals). The aim of this review is both to summarise the present European legislation on depositions both to discuss the different legislations adopted by the various member states. Furthermore, a focus of this paper will be dedicated to the Italian legislation, where there is currently no specific guideline values for POPs in atmospheric deposition. In any case, some national authorities in Italy, such as the National Institute of Health (ISS) and the Regional Environmental Protection Agencies (ARPA), have conducted numerous monitoring activities on depositions, providing the scientific community and policymakers with numerous data on which to establish national reference values.

## Introduction

Many of the pollutants emitted by anthropogenic and natural sources present on the particulate matter are removed from the atmosphere through deposition (Omrani et al. 2017). This phenomenon is known as total or bulk deposition (BD) and refers to ‘the total mass of pollutants that is transferred from the atmosphere to surfaces (e.g. soil, vegetation, water, and buildings) in a given area within a given period of time’ (Parliament and European Council 2004a). The term ‘bulk’ means that atmospheric depositions are the sum of wet and dry fraction. Wet deposition is when pollutants fall to the ground through the action of atmospheric precipitation (rain and snow), while wet deposition refers to the sedimentation of pollutants under the action of gravity (Bisquert et al. 2017). BD measurements are useful for verifying the state of contamination of an area with regard to POPs and inorganic micropollutants (metals and metalloids) that tend to accumulate in the environment (Settimo and Viviano 2015). In particular, they are essential for assessing of the levels of deposition on the ground of the most harmful substances for human health such as polychlorinated dibenzo-p-dioxins (PCDDs) and polychlorinated dibenzofurans (PCDFs), polychlorinated biphenyls dioxin-like compounds (DL-PCBs), polycyclic aromatic hydrocarbons (PAHs), and metals (nickel, lead, cadmium, etc.). Deposition rate can be considered a way of indirect exposure to such pollutants through diet. In fact, according to the World Health Organisation (WHO), a high contribution to the total intake of PCDD/Fs is through food consumption (World Health Organization 2000). Even about the intake of benzo[a]pyrene and PAHs, contaminated food consumption is the main source of exposure (European Commission 2001a). During the past 40 years, the topic of atmospheric deposition has been repeatedly addressed by international authorities and programmes. Starting with the 1979 international Long-RangeTransboundary Air Pollution Convention (CLRTAP) and the subsequent Co-operative Programme for Monitoring and Evaluation of Long-Range Transmission of Air Pollutants in Europe (EMEP), the international community strongly suggested the adoption of monitoring activities for PCDD/Fs, PCBs, PAHs, and metals in BD (United Nations Economic Commission for Europe 1979; European Community 1986). The aim was to increase knowledge about the role that depositions play in terms of health impacts. In 1992, the Oslo and Paris Convention for the Protection of the Marine Environment of the North-East Atlantic (OSPAR) (n.d) also highlighted the need to implement deposition information on the main persistent pollutants (European Community 1992). At the same time, the United Nations Economic Commission for Europe (UNECE) through the signing of the Aarhus Protocol in 1998, aims to protect the health of the population by requiring member states to make public data and information on environmental pollution (monitoring of POPs deposition flux is also included in this data) (Mason 2010). The Aarhus Protocol refers to a number of persistent organic substances that need to be eliminated or their emissions reduced but does not directly mention atmospheric depositions. In 2001, under the request of the United Nations Environment Programme (UNEP), the Stockholm convention was adopted, and the importance of monitoring the atmospheric deposition of major POPs in soil and ecosystems was emphasised (Lallas 2001). A document of the Food and Agriculture Organisation of the United Nations (FAO) also puts in evidence the great contribution that the deposition of metals and metalloids, PAHs, PCDD/Fs, and pesticides has in soil and plant contamination (Food and Agriculture Organization 2018). The subsequent passage of POPs into agricultural products (mainly, fruit and vegetables), fodder, and consequently, into animals is well known and documented by multiple studies in literature, where the population is exposed to these substances through the consumption of contaminated foodstuffs (Florence et al. 2015; Zhang et al. 2017; Youssef and Abd El-Gawad 2018; Luo et al. 2020; de Pinho et al. 2023; Kiani et al. 2023; Fiolet et al. 2024). For this reason, it is important to monitor total deposition, especially in areas where industrial activities such as incinerators, storage wastefires, and thermoelectric coal plants are located, in order to assess the accumulation of POPs in the soil and to evaluate the possible subsequent effects on human health (Die et al. 2021). Although the determination of deposition fluxes is a good environmental surveillance system, very few studies on this topic have been conducted in the scientific literature in recent years (Settimo and Viviano 2015). In fact, this can be evidenced by a literature search on the *Scopus* database: the authors, using the keyword ‘atmospheric deposition’, and setting the search over the last 5 years (from 2017 to 2022), obtained a total of 173 publications. Despite the small amount of scientific work on the subject of depositions, legislators in some European countries have nevertheless adopted different recommendations, guidelines, and limit values to be respected, in particular for PCDD/Fs, DL-PCBs, and some metals. In this context, the aim of this paper is to summarise the European legislation on BD, highlighting the differences between the limit values or guideline values adopted by the different member countries. In addition, the authors will devote a paragraph to Italy, describing the legislation, the main papers present in literature, and the technical-scientific reports produced by various National bodies such as National Institute of Health (ISS) and Regional Environmental Protection Agencies (ARPA).

## The main European legislation on atmospheric bulk depositions

Since 2003, the European Union (EU) has adopted a series of legislative acts aimed at minimising the release of POPs and stopping their manufacture, marketing, and use in order to protect the health of the population and the environment (Parliament and European Council 2003, 2004b). The main act was the approval and implementation of the Stockholm convention on POPs through Council Decision 2006/507 (European Council 2004). The focus was placed on a list of 12 ‘priority’ substances, including PCDD/Fs and PCBs. On the basis of these acts and the evidence of the effects that these molecules generate on human health, the European Commission through an initial communication stresses the importance of the study on the deposition of PCCD/Fs and PCBs. With this communication, the depositions begin to be attentively studied; in fact, the legislator writes that in order to ‘limit human intake of these substances, it is important to reduce their levels in the food chain’. For example, dioxins present in the air can be deposited on plants or in water and from there pass into animals and fish through food, thus entering the food chain (European Commission 2001b, 2004b, 2007). Through these legislative acts, also known as ‘community strategy on dioxins, furans, and polychlorinated biphenyls’, the EU has defined several objectives and strategies, which are described below:

- Reduce the presence of PCDD/Fs and PCBs in the environment
- Reduce the quantities of PCDD/Fs and PCBs in food and feed
- Reduce human exposure to PCDD/Fs and PCBs in the short term and maintain human exposure at safe levels in the medium to long term
- Reduce human intake levels below 14 pg WHO-TE/kg body weight per week

The reduction of the amounts of PCDD/Fs and PCBs in food and feed is strongly dependent on the amounts of these substances in the environment and the contribution of their atmospheric deposition to the soil (Food and Agriculture Organization 2018). It is well known that the main mechanism of transfer of POPs from the atmosphere to the soil and vegetation is total deposition (Jimenez et al. 2015). In this context, the European commission, through its 2001 Communication in Annex III, highlights the importance of measuring the deposition of PCDD/Fs and PCBs in monitoring and exposure assessment activities. Also in Directive 2004/107/EC, concerning arsenic (As), cadmium (Cd), mercury (Hg), nickel (Ni), and PAHs in ambient air, the legislator put in evidence that adverse health effects are due not only to the concentrations of these molecules in ambient air but also to their deposition on the soil, which allows the passage and accumulation in the food chain of persistent organic substances. Generally, during environmental monitoring, there is a tendency to give exclusive attention to the effects that suspended particulate matter (SPM) generates on the health of exposed people, forgetting the indirect contribution that deposition makes through the ingestion of contaminated food. It is therefore essential, during monitoring and subsequent risk assessment activities, to consider the contribution to exposure through ingestion due to the deposition of settleable particulate matter (PM). This is well highlighted in Directive 2004/107/EC, which urges member states to promote research into the effects of arsenic, cadmium, mercury, nickel, and PAHs on human and environmental health, in particular through deposition (Parliament and European Council 2004a). In order to carry out and facilitate research on the role of depositions, it is necessary to provide member states and competent authorities with common criteria for the location of measuring stations, suitable deposition collection systems, and standardised analytical protocols, so that the information obtained can be compared across European countries. On this aspect, both Directive 2004/107/EC and the subsequent Directive 2008/50/EC have entrusted the European Standardisation Committee with the elaboration and development of CEN standard methods for the determination of the main POPs present in BD (Parliament and European Council 2008). With regard to the location of measurement stations, these must be chosen by member states in such a way as to provide deposition rate data representing the indirect exposure of the resident population through diet. For example, Italy, through Ministerial Decree 29/11/2012 (Republic of Italy, 2012a), to increase knowledge on the role of BD, has identified and set up four stations to measure total As deposition, Cd, Ni, Hg, benzo(a)pyrene, and other PAHs of toxicological relevance, including benzo(a)antracene (BaA), benzo(b)fluoranthene (BbFA), benzo(j)fluoranthene (BjFA), benzo(k)fluoranthene (BkFA), indeno(1,2,3-cd)pyrene (INP), and dibenzo(a,h)antracene (DBahA). These stations were distributed to obtain representative information on ground deposition rates. Concerning the collection system and analytical protocol to be used for the determination of metals of hygienic interest and PAHs in BD, Directive 2015/1480/EC on ‘reference methods and validation of data’, indicates the CEN standard to be used, specifically EN 15841:2010 (European Commission 2015). Other European standards for the determination of micropollutants in BD were developed in the same years. Table 1 lists all the official standards already issued.

**Table 1:** CEN standards already published for the sampling and analysis of organic and inorganic micropollutants in atmospheric depositions

CEN standard	Title	References
EN 15980:2011	Air quality. Determination of the deposition of benz[a]anthracene, benzo[b]fluoranthene, benzo[j]fluoranthene, benzo[k] fluoranthene, benzo[a]pyrene, dibenz[a,h]anthracene and indeno[1,2,3-cd]pyrene	(European standards committee 2011)
EN 15841:2010	Ambient air quality — Standard method for determination of arsenic, cadmium, lead and nickel in atmospheric deposition	(European standards committee 2010a)
EN 15853:2010	Ambient air quality — Standard method for determination of mercury deposition	(European standards committee 2010b)
EN 1948–2:2006	Stationary source emissions—Determination of the mass concentration of PCDDs/PCDFs and dioxin-like PCBs—Part 2: Extraction and clean-up of PCDDs/PCDFs	(European standards committee 2006a)
EN 1948–3:2006	Part 3: Identification and quantification of PCDDs/PCDFs	(European standards committee 2006b)
EN 1948–4:2014	Part 4: Determination of the mass concentration of PCDDs/PCDFs and dioxin-like PCBs—Part 4: Sampling and analysis of dioxin-like PCBs (includes Amendment A1:2013)	(European standards committee 2014)

**Fig. 1:**
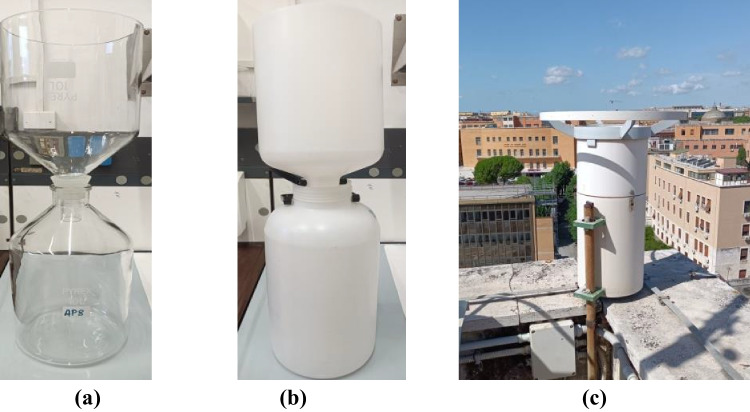
**a** Glass deposimeter for organic pollutants sampling in BD; **b** HDPE deposimeter for inorganic pollutants sampling in BD; **c** total bulk deposimeter sampler

The deposition collection system is a passive system, called deposimeter. The type of deposimeter that is used during sampling depends on the type of deposition that is to be collected (BD or wet only deposition). The deposimeter for collecting BD consists of a ‘bottle + cylindrical funnel’ system, with a wet deposition collection capacity of 10 l. In contrast, the deposimeter for the collection of the wet-only fraction comprises only a collection bottle and a specific sensor that allows it to be opened or closed according to the presence or absence of precipitation. This is used in countries with high rainfall (rain and snow) that do not allow optimal sampling. In addition, wet-only sampling may be used instead of bulk sampling if it can be shown that the difference between their results is within 10%. A further distinction in sampling lies in the material of which the deposimeter is made, as high-density polyethylene (HDPE) deposimeters are used for the collection and determination of the inorganic fraction (European standard committee 2010a), while Pyrex® glass deposimeters are used for the organic fraction (European standard committee 2011). For both, sampling must have a minimum exposure time of 1 week and a maximum of 1 month. Sampling must therefore be evenly distributed throughout the year to assess seasonal variations and long-term trends. Of the two systems, the one for collecting BD is shown to be better suited to providing reproducible and robust results, allowing important information to be obtained on the contamination status of a specific area. For clarity, the authors show in Fig. 1a, b, and c, respectively, a Pyrex® glass deposimeter, an HDPE, and one prepared for field sampling equipped with a protective structure for exposure to the sun and atmospheric agents.

At the end of sampling, for both deposimeters, the collected aqueous portion is filtered through a membrane filter (glass fibre filter for organics and cellulose filter for inorganics) and the particulate component deposited on the filter is weighed to calculate the amount of PM collected. The extraction of organic micropollutants from the PM collected on the filter and from the wet deposition is performed using a suitable organic solvent in an ultrasonic bath and separating funnel (liquid–liquid-extraction, LLE), respectively. Instrumental analysis is conducted by gas chromatography (GC) with a single or triple quadrupole mass spectrometry (qMS or MS/MS) detector. Metals, on the other hand, are determined following mineralisation of the filter and filtrate in a solution of concentrated nitric acid and hydrogen peroxide. The final aqueous solution is subjected to analysis in GFAAS or ICP/MS. The introduction of both Directive 2004/107/EC (Parliament and European Council 2004a) and the CEN methodological standards (reported in Table 1), providing a common basis for action, has encouraged member states to implement atmospheric deposition monitoring campaigns, increasing the knowledge about environmental and health effects (Settimo and Viviano 2015). Over the years, this has led to the adoption by some European countries of limit values for depositions of the major compounds harmful to the health of the population, including PCDD/Fs, DL-PCBs, and metals. At present, no legal limits or guideline values have been set at the European level for pollutant depositions, but legislation has been issued that only urges member states and the scientific community to increase studies and assessments of the health impacts of total depositions. In Table 2, the authors summarise the European regulatory acts that focus on the issue of BD. The legislative acts listed do not indicate any kind of guideline value for the deposition of POPs, leaving the member states free to adopt national laws in which they indicate limit/guide values to be respected within their territory. This cannot justify the absence of dedicated European legislation, which is in any case necessary to give due importance to the issue of BD.

**Table 2:** List of European regulatory acts on BD

Regulatory act	Year	Title	References
COM (2001) 593	2001	Communication from the commission to the council, the parliament and the economic and social committee. “Community strategy on dioxins, furans and polychlorinatedbiphenyls”	(European Commission 2001a, b)
Directive 2004/107/CE	2004	Directive “relating to arsenic, cadmium, mercury, nickel and polycyclic aromatic hydrocarbons in ambient air”	(Parliament and European Council 2004a, b)
COM (2007) 396	2007	“Community strategy on dioxins, furans and polychlorinated biphenyls”- second summary report	(European Commission 2004)
Directive 2008/50/CE	2008	Directive “on ambient air quality and cleaner air for Europe”	(European Commission 2008)
COM (2010) 562	2010	“Community strategy on dioxins, furans and polychlorinated biphenyls”- third summary report	(European Commission 2007)
Directive 2015/1480/CE	2015	Directive “amending several annexes to Directives 2004/107/EC and 2008/50/EC of the European Parliament and of the Council laying down the rules concerning reference methods, data validation and location of sampling points for the assessment of ambient air quality”	(European Commission 2015)

## The new proposal for a European air quality directive and the role of atmospheric bulk deposition

The authors felt it was important to devote a paragraph to the European Commission’s recent proposal to introduce a new directive on ambient air quality and to identify the salient points on the subject of atmospheric deposition. The proposal of the new directive, made through the communication ‘COM (2022) 542/final 2’ (Parliament and European Council 2022) and the latest ordinary legislative procedure (European Council 2024), is one of the key actions envisaged in the European Green Deal and the Action Strategy to achieve zero pollution for air, water and soil (European Commission 2019). Briefly, the pillars on which the proposal is based can be summarised as follows:

- To ensure that European laws on ambient air strictly follow the WHO recommendations and guidelines on air quality
- Provide support to local authorities in achieving the goal of cleaner air by strengthening air quality monitoring and plans
- Provide a single legislative framework for ambient air that emphasises the protection of the health of the population and provides adequate information to the public

Within the document, despite the fact that deposition rates are not yet regulated, the legislator pays strong attention to BD issue, reiterating their contribution to human exposure to carcinogens through the food chain (indirect route). Among the substances harmful to human health present in depositions, the new directive also includes lead (Pb) in addition to As, Cd, Hg, Ni, and PAHs. In order to enable a better understanding of the effects of the deposition of these substances on human health, the directive in article 10 requires all member states to carry out monitoring at urban and rural sites distributed throughout the national territory. BD monitoring plans must be adopted, where in order to protect human health (especially for the most vulnerable groups of people, including children and the elderly), it is essential to provide data not only for dispersed air pollutants but also for BD. These are set out in Annex IV on the assessment of ambient air quality and the location of sampling points. Other clarifications and changes are made, including a change in the minimum data coverage to be provided by authorities on monitoring activities. Specifically, Annex V specifies that for deposition, at least 30% of annual average measurements must be covered. In conclusion, this proposal for a directive, by highlighting the important contribution that BD have on the exposure of the population to POPs, may lead to a greater knowledge of BD and to the consequent adoption of limit values that take into account the health effects that the depositions have on the population health.

## Activities conducted in various European countries on BD

Guide values and limit values for BD and pollutants were adopted by the various member states taking into account the potential ingestion of contaminated foodstuffs. Twenty years ago, the WHO specified in several documents that for PCDD/Fs and PCBs, indirect exposure through food consumption represented the major route of human exposure (World Health Organization 2002). In recent years, due to reduced concentrations of PCDD/Fs and PCBs in all environmental matrices, inhalation and dietary intake both contribute significantly to total daily intake-TDI (IARC 2015; World Health Organization 2019). In fact, PCDD/Fs and PCBs in air can be a significant source of exposure, especially near to local emissions, such as industrial plants (IARC 2015; Luarte et al. 2023). However, given the consistent dietary contribution to the intake of PCDD/Fs, DL-PCBs, and PCBs and the inherent characteristics of these substances to accumulate in animal fats, various authorities worldwide and in Europe have introduced tolerable intake on a daily (TDI), weekly (TWI), and monthly (TMI) basis. These limits refer to intake through the ingestion of contaminated foods (mainly high-fat foods of animal origin and dairy products) due mainly to the deposition on soil and plants of POPs with subsequent passage through the food chain. Following studies and monitoring, in 1998, the WHO established a cumulative value for the TDI of PCDD/Fs and DL-PCBs in the range of 1–4 picograms (pg) toxic equivalent (WHO-TE) per kilogram of body weight (b.w). In 2001, the WHO, through the Joint Expert Committee on Food Additives (JECFA), in collaboration with FAO, introduced another limit value, related to the TMI for PCDD/Fs and DL-PCBs of 70 pg/kg b.w (World Health Organization 2002). In the same year, a cumulative value for the TWI for PCDD/Fs and DL-PCBs of 14 pg WHO-TE/kg b.w was established at European level by the Scientific Committee on Food (SCF) (Scientific Committee on Food 2001). Different authorities have expressed tolerable doses on different exposure time bases, but by converting all limits to a TDI, the latter are assimilated. In 2018, the European Food Safety Authority (EFSA) reduced the TWI value by seven times, from 14 to 2 pg WHO-TE/kg b.w (EFSA 2018). The reasons for the reduction lie in the availability of new models for estimating the accumulation of dioxins in the human body and from evidence in exceeding tolerable doses in all age groups (EFSA 2018). This represents an important value to be used in exposure assessment pending reviews of toxicity equivalent factors for PCDD/Fs and DL-PCBs. Table 3 shows the EFSA updated TWI value for PCDD/Fs and DL-PCBs and the last value proposed by SCF in 2001 highlighting the consistent reduction in tolerable dose.

**Table 3:** Current health-based limit values established by different decision-making organisation and their value converted in TDI

Decision-making organisation and year	Health-based limit values in pg WHO-TE/kg b.w	Value converted to TDI in pg WHO-TE/kg b.w	References
EFSA (2018)	TWI 2	0.3	(EFSA 2018)
SCF (2001)	TWI 14	2	(Scientific Committee on Food 2001)

Some European countries have calculated guide and limit values for BD from the TDI values. These values were calculated using modelling software taking into account the following variables: (a) calculation of BD, (b) estimation of the amounts of contaminants in BD, (c) transfer of substances in the food chain and estimation of dietary intake. For example, Germany and Belgium have proposed guideline values for PCDD/Fs and DL-PCBs deposition in relation to TDI values established by the WHO and SCF. Table 4 shows the derivation of the guideline values for PCDD/Fs and DL-PCBs depositions from those of the TDI.

**Table 4:** Proposed guideline values for PCDD/Fs deposition in relation to different TDI

TDI pg WHO-TE/kg b.w	Deposition of PCDD/Fs pg WHO-TE m^−2^ day^−1^ (annual average)	Deposition of PCDD/Fs pg WHO-TE m^−2^ day^−1^ (monthly average)	References
1	3.4	6.8	(Van Lieshout et al. 2001)
2	8.2*	21*	(Desmedt et al. 2008)

^*^DL-PCBs were also included in the calculation of deposition values

Some deposition guide values were then adopted as limit values on annual and/or monthly basis. This aspect will be discussed by the authors in next sections: limit values for different POPs in BD will be reported and compared for some EU Member states.

### Germany

In Germany during the last 20 years, BD legislation has been enacted: since 2002, through the introduction of the *Technische Anleitungzur Reinhaltung der Luft*-TA Luft, a PM limit value of 350 mg m^−2^ day^−1^, calculated as annual average, has been established (Republic of Germany 2002). In addition, a PM value of 650 mg m^−2^ day^−1^ is also given when calculated as a monthly average. In the 2004 update of the TA-Luft, the German legislator, starting from a TDI value of 2 pg WHO-TE/kg b.w, proposes a maximum permissible deposition limit for PCDD/Fs and DL-PCBs in the range of 1.1 to 5.5 pg WHO-TE m^−2^ day^−1^ as annual average (Republic of Germany 2004). Based on a study related to the limitation of deposition on pasture and forage fields, aimed at reducing the indirect intake of dioxins through meat and dairy products, a guideline value of 4 pg WHO-TE m^−2^ day^−1^ was set for the deposition of PCDD/Fs and DL-PCBs. Instead, a BD limit value for PCDD/Fs and DL-PCBs of 9 pg WHO-TE m^−2^ day^−1^ was set for industrial plants. In subsequent years, specific limits for the deposition of certain metals (As, Cd, Hg, Ni, Pb, and Tl) have been introduced, giving precise indications for sampling with the *Bergerhoff* system (Germany, *Verein Deutscher Ingenieure* 2012). In countries where wet and dry deposition rates are high, it is possible to use the *Bergerhoff* collection system, which allows only the dry residue of total depositions to be measured, obtaining results equivalent to other collection systems (Thöni et al. 1999). Furthermore, this system can be used for monitoring deposition in urban areas with high car traffic or in the surroundings of industrial plants where the dry deposition component is significant (Settimo and Viviano 2015; Republic of Germany 2021). Metal deposition is expressed as an annual average in µg m^−2^ day^−1^. Finally, through the most recent revision of the TA-Luft of 18 August 2021, a limit value of 0.5 µg m^−2^ day^−1^ (annual average) was introduced for the first time for the deposition of benzo[a]pyrene, the main compound used as an index of carcinogenicity of PAHs in a given food and/or environmental matrix (Republic of Germany 2021). Table 6 summarised the current guide and limit values for depositions in Germany.

### Belgium

In 1995, the Belgian government issued the first piece of legislation in which guide and limit values for sedimentable particulate matter deposition were introduced. Specifically, a guide value for PM of 350 mg m^−2^ day^−1^ on an annual average was suggested (Belgium government 1995). At the same time, a limit value of 650 mg m^−2^ day^−1^ PM on a monthly average was established. The legislator also specifies that BD monitoring activities must be carried out in the vicinity of industrial plants, respecting the imposed limit value for PM (Belgium government 1995). In the same years, the Flemish Environment Agency (VMN) and the Flemish Institute for Technological Research (VITO) initiated monitoring campaigns of atmospheric depositions of PCDD/Fs throughout Belgium with the aim of assessing the contribution of these substances’ depositions on the total exposure of the population. These studies initially proposed a guideline value for PCDD/Fs BD of 10 pg WHO-TE m^−2^ day^−1^ (annual average) (Van Lieshout et al. 2001). Since 2002, the Belgian agencies have also included the determination of DL-PCBs in BD and in particular PCB-126 in their monitoring programme. It was therefore necessary to update the proposed guide value of 10 pg WHO-TE m^−2^ day^−1^ by adding DL-PCBs in the calculation of the new value. Considering the correlation between the TDI value of 2 pg WHO-TE/kg b.w and the PCDD/Fs and DL-PCBs deposition data obtained from monitoring activities, a new guide values of 8.2 pg WHO-TE m^−2^ day^−1^ (annual average) and 21 pg WHO-TE m^−2^ day^−1^ (monthly average) were proposed (Desmedt et al. 2008).With regard to metals, only for Cd and Pb were limit values established. Finally, for PAHs in BD, no guideline value was suggested and introduced in the Belgian legislation. Table 6 summarised the current guide and limit values for depositions in Belgium.

### France

In France, the regulatory framework for atmospheric deposition is extremely deficient. Very few BD monitoring activities have been conducted and only for PCDD/Fs. No works and no data are available for metals and other POPs. In 2001, the Institut National de l’Environnement Industriel et des Risques (INERIS), in a document entitled ‘Méthode de surveillance des retombées des dioxines et furanes autour d’une unite d’incinération des ordures ménagères (UIOM)’, emphasises the importance of deposition in the contamination of soil and subsequently dairy products (INERIS 2001). INERIS in this document establishes a maximum limit of PCDD/Fs concentration in milk of 5 pg I-TE/g of fat and sets a target value of 1 pg I-TE/g of fat. The latter value, even when calculated using International toxicity factors (I-TE, NATO/CCMS 1988) (Kutz et al. 1990), tend to be in line with the 2 pg WHO-TE/g of fat value in the Regulation 2022/2002/UE (European Commission 2022). Starting from PCDD/Fs concentration values between 1 and 4 pg I-TE/g of fat, the French agency estimated a maximum PCDD/Fs BD to soil of 40 pg I-TE m^−2^ day^−1^ (annual average) to meet the maximum permitted levels in dairy products (Albinet 2015). This value is considerably higher than those proposed by Belgium and Germany but reflects the state of contamination on French territory. Indeed, in Durif (2001), it is specified that in some areas, the level of 40 pg I-TE m^−2^ day^−1^ may be much lower than the background pollution. Some studies, on the other hand, have proposed PCDD/Fs BD values to be used to monitor the state of air quality near incineration plants (Bodénan et al. 2011). Specifically, it has been suggested that PCDD/Fs deposition values of less than 5 pg I-TE m^−2^ day^−1^ are representative of the background noise of urban-industrial areas, while values between 5 and 16 pg I-TE m^−2^ day^−1^ indicate that the area where monitoring was carried out is influenced by anthropogenic activities that increase the average background levels of PCDD/Fs. Finally, where deposition values exceed 16 pg I-TE m^−2^ day^−1^, the proximity of an emission source is evident. In this case, the authorities in charge must increase monitoring activities at several points and determine the congeners profile in order to identify the specific emission source and take elimination and/or mitigation actions. These values were updated in 2012 by INERIS based on the monitoring of PCDD/Fs BD in a French incineration plant from 1991 to 2012 (INERIS 2012). Table 5 summarises the latter guideline values used by the French authorities for monitoring PCDD/Fs BD.

**Table 5:** Guide values for PCDD/Fs depositions in pg I-TE m^**−**2^ day^**−**1^ (annual average) (INERIS 2020)

Type of site	Average	Median
Rural background	1.7	1.6
Urban background	3.0	2.0
More than 500 m below the wind direction from the incinerator	2.8	2.1
Between 100 and 500 m below the wind direction from the incinerator	3.6	3.3
Less than 100 m below the wind direction from the incinerator	15.7	6.9

In conclusion, INERIS in a 2021 document entitled ‘Guide sur la surveillance dans l’airautour des installations classées—Retombées des emissions atmosphériques’, specifies that for some substances such as Pb, Cd, Ni, Hg, and PAHs, it is necessary to monitor not only their presence in ambient air but it is essential to measure their quantities in atmospheric BD (INERIS 2021). The document summarises the methodologies already in place for sampling and analysing BD (LCSQA 2011, 2015), emphasising the need to adopt a regulatory framework to regulate the main POPs in BD.

### Croatia

Croatia, together with Germany, is one of the European countries with the highest number of regulated micropollutants in atmospheric BD. In 2005, the Croatian government issued a regulation listing the permitted limit values for pollutants in the air, including total depositions (Republic of Croatia 2005). Briefly, the limit value for PM was set at 350 mg m^−2^ day^−1^ (annual average), in line with the German and Belgian value. For metals, the set values are also identical to the German values, with the exception of the depositions of organic contaminants (PCDD/Fs, PCBs and PAHs) where no limit value or guide value was set. On this issue, the Croatian government should increase monitoring activities in order to obtain more data that can be used to establish and introduce limits for the BD of organic micropollutants. See Table 6 for the BD current limit values in Croatia.

**Table 6:** Current guidelines and limit values (annual average) in European countries for BD; PM (mg m^−2^ day^−1^), PCDD/Fs + DL-PCBs (pg WHO-TE m^−2^ day^−1^), benzo[a]pyrene(µg m^−2^ day^−1^), and metals (µg m^−2^ day^−1^) in BD

	PM	PCDD/Fs + DL-PCBs	BaP	As	Cd	Hg	Ni	Pb	Tl	Zn
Germany	350 650*	4^GV^	0.5	4	2	1	15	100	2	-
Belgium	350^GV^ 650*	8.2^GV^ 21*^GV^	-	-	2	-	-	250	-	-
Croatia	350	-	-	4	2	1	15	100	2	-
Austria	210	-	-	-	2	-	-	100	-	-
UK	200	-	-	-	-	-	-	-	-	-
Switzerland	200	-	-	-	2	-	-	100	2	400
Slovenia	200	-	-	-	2	-	-	100	-	400

^*^Monthly average, ^GV^guide value

### Summary of BD guide/limit values in European countries

In addition to the countries described above, Austria, United Kingdom, Switzerland, and Slovenia have also introduced limit values in their legislation for BD, especially for PM and metals (Switzerland 1985; Slovenia 1994; Republic of Austria 1997; Environment Agency UK 2013). Table 6 summarises all the guide/limit values for BDs in the European countries mentioned. To date, no other European country has introduced limit values for BD.

### BD monitoring activities in various European countries

Multiple monitoring campaigns and reports always show PM values below the legal limits (Wallenhorst et al. 1997; Bisquert et al. 2017; Nežiková et al. 2019; Dufour et al. 2021; ARPAL 2023), highlighting the fact that during the warm season (spring and summer), the values of settleable dust are much higher than during the cold season (autumn and winter). This is explained by the lower precipitation and the phenomenon of dust rising from the ground (Settimo et al. 2021). Values of PCDD/Fs in atmospheric deposition found in different parts of Europe are highly variable. In all countries, higher levels of PCDD/Fs in BD are found in urban areas than in rural ones (Dufour et al. 2021; Dreyer and Minkos 2023). The contribution of specific sources in the urban environment, such as road transport, industrial and non-industrial combustion and the use of domestic heating systems during the winter months, is evident (Gunes and Saral 2014; Santa-Marina et al. 2023). The values of PCDD/Fs in BD found in rural areas in different European countries tend to be in line with the guideline values of 4 and 8.2 pg WHO-TE m^**−**2^ day^**−**1^ adopted by Germany and Belgium, respectively. The situation is different for urban and industrial areas, where high levels are reached due to the contribution of industrial production activities. Table 7 summarises the levels of PCDD/Fs in BD in some European countries.

**Table 7:** Comparison of PCDD/Fs values (pg I-TE m^−2^ day^−1^ annual average) in BD in different European areas and countries

Country	Rural sites (min–max)	Urban sites (min–max)	Industrial sites (min–max)	References
Belgium	< 1–3.1	< 1–12	1–211	(Van Lishout et. 2001; Desmedet et al. 2008; Dufour et al. 2021)
Germany	7–17	0.5–464	-	(Kirchner et al. 2020; Dreyer and Minkos 2023)
United Kingdom	< 1–517	< 1–312	2–118	(Buckley-Golder et al. 1999)
Denmark	0.5–31.5	1.7–31.6	-	(Vikelsøeet al. 2005)
France	20–50	100–147	4.2–363*	(Castro-Jiménez et al. 2011; Meyer et al. 2015)
Finland	0.1–3**	-	-	(Korhonenet al. 2016)
Sweden	< 0.1–0.7	-	-	(Sellström et al. 2009)

^*^Values are calculated using WHO-TEF_1998_

**values are calculated using WHO-TEF_2005_

With regard to PAHs in BD, much monitoring work has been conducted in many European countries. Unlike the other pollutants, no guide values are given for PAHs in BD, apart from the value of 0.5 µg m^**−**2^ d^**−**1^ in Germany related to BaP. In various monitoring works carried out in different rural sites, the BaP BD limit value proposed by Germany is never exceeded. In several European urban areas, BaP values in BD are also below 0.5 µg m^**−**2^ day^**−**1^, with the exception of a maximum value of 2.2 µg m^**−**2^ day^**−**1^ reported in (Halsall et al. 1997). The literature shows the higher concentrations of PAHs in deposition in urban centres compared to rural and remote areas. This is due to the contribution of urban emission sources, such as vehicle traffic and the operation of domestic heating systems (Amodio et al. 2014). Table 8 lists the values of BaP in BD found in several European countries.

**Table 8:** Comparison of BaP values (ng m^−2^ day^−1^ annual average) in BD in different European areas and countries

Country	Rural sites (min–max)	Urban sites (min–max)	References
Germany	4.4–31.8	-	(Gocht et al. 2007)
United Kingdom	-	36.2–2220	(Halsall et al. 1997)
Poland	-	19.8–396	(Siudek 2022)
France	0.1–34.1	0.1–252	(Motelay-Massei et al. 2007; Ollivon et al. 2002)
Finland	2–10	-	(Korhonen et al. 1998)
Greece	0.3–3.5	0.9–17.5	(Terzi and Samara 2005)
Sweden	5–17	-	(OSPAR Commission 2001)

With regard to the assessment of metal content in BD, the European Commission in a 2001 paper summarises several scientific works aimed at monitoring the inorganic component in depositions (European Commission 2001b). Table 9 shows the content of As, Cd, and Ni in BD detected in European countries at sites with different anthropogenic impacts. Taking the limit values established in the various countries as a reference, the data show that only industrial sites have values of As, Cd, and Ni that exceed the imposed limits. In fact, it is well known that in most cases, the high levels of metals occur in the close vicinity of the industrial production building (for example: incinerators and storage waste fires, iron and steel plants and thermoelectric coal plants) and then decrease as one moves away from the emissive source (Flemish Environmental Society, 2015). This is mainly due to the contribution of coarse particles that are deposited on the ground with high velocity (Bergametti et al. 2018). In conclusion, the presence of metals in depositions is predominant at industrial sites, where BD monitoring activities must be constantly conducted to provide information on the site’s contamination status.

**Table 9:** Comparison of As, Cd and Ni values (µg m^−2^ day^−1^ annual average) in BD in different sites in Europe

Metals	Type of site			References
	Rural	Urban	Industrial	
As	0.082–0.43	0.22–3.4	2.0–4.3	(European Commission 2001b)
Cd	0.011–0.14	0.16–0.90	0.12–4.6	
Ni	0.030–4.30	5–11	2.3–22.0	

## The Italian situation

In Italy, the legislative framework on BD does not provide any guide or limit value for any type of micropollutant. Despite the absence of reference values, a series of legislative acts have been issued over the years that in different ways highlight the importance of studying BD and their effect on human health and the environment. Beginning in 1966 with Law no. 615 ‘Measures against Atmospheric Pollution’, which placed particular attention on the atmospheric emissions of fumes, dust, and gases, the legislature established a ‘Central Commission against Atmospheric Pollution’ to promote studies and research on pollutants emitted into the atmosphere, including dust (Republic of Italy 1966). In 1983, this Commission proposed reference values for settleable dust in order to classify the territorial areas where monitoring was carried out with specific dustiness classes. Table 10 shows the dust classes and the corresponding annual average values of settleable dust in mg m^**−**2^ day^**−**1^.

**Table 10:** Dust classes and indices with respective sediment dust values in mg m^−2^ day^−1^ as annual average

Classes	Sediment dusts (mg m^−2^ day^−1^)	Dustiness index
I	< 100	Virtually absent
II	100–250	Low
III	251–500	Medium
IV	501–600	Medium–high
V	> 600	High

Dustiness indices have been and still are used by environmental authorities (ARPA) as a reference for assessing dust levels, especially in industrial areas. The need to adopt atmospheric deposition detection networks in order to protect public health in sites with different anthropic impact (remote, rural, urban, and industrial) was reaffirmed in the subsequent Decree of the Ministry of the Environment and Health of 20 May 1991 on the ‘criteria for the collection of air quality data’. This is stated in Annex I, paragraph 1.6 Non-automatic measures – Species to analyse, where it is specified that atmospheric deposition can be of the dry and wet type. Furthermore, the difference between dry deposition and settleable dust is explained (Republic of Italy 1991). Dry deposition is significant in industrial areas and comprises the fine fraction (< 0.1 μm) (European Commission 2001a, b), while settleable dust consists of the particulate material with a very high particle size and which settles under the action of the gravity field. Different types of chemical analysis can be performed on the deposited dust. In the following years, Italy implemented European Directives 2004/107/EC and 2008/50/EC through the enactment of Legislative Decree (D.Lgs) no. 152 of 2007 and D.Lgs no. 155 of 2010, respectively (Republic of Italy 2007, 2010). In D.Lgs 152/2007, in addition to the definition of ‘total deposition’-BD, the legislator sets out to define sampling and analysis methods and criteria for assessing the deposition of As, Cd, Hg, Ni, and PAHs. The subsequent D.Lgs 155/2010 firstly repeals the Ministerial Decree of 20 May 1991 and D.Lgs 152/2007 and secondly places a strong emphasis on obtaining information on air quality, including the monitoring of BD, in order to identify measures to reduce harmful effects on human health. In fact, in art. no. 6 ‘special cases of ambient air quality assessment’, in order to monitor the trend of BD over time and their effects on health, at least three national background measurement stations must be identified. Through the Ministerial Decree of 29 November 2012 (Republic of Italy 2012a), four special monitoring stations were identified for measuring the BD of As, Cd, Hg, Ni, BaP, and other PAHs of toxicological relevance, including BaA, BbFA, BjFA, BkFA, INP, and DBahA. For the monitoring of As, Cd, Ni, and PAHs, the following stations were chosen: Schivenoglia in the province of Mantua, Ripatransone in the province of Ascoli Piceno, and Monte Sant’Angelo near Foggia. All stations were classified as rural background stations. Instead, for monitoring total Hg deposition, the municipality of Montelibretti in the province of Rome was chosen as the station, classified as a background station in a suburban site. Furthermore, D.Lgs 155/2010 provides in detail in Annexes I and VI the duration of sampling (minimum 1 week and maximum 1 month), the minimum time period of monitoring (annual basis), and the reference methods for measuring the BD rates of As, Cd, Ni, and PAH, indicated in the ISTISAN Report 06/38 produced by ISS in 2006 (Menichini et al. 2006a). Annex III instead reiterates the importance of the monitoring data of BD as they are useful for evaluating the indirect exposure of the population to pollutants through the food chain. Finally, Legislative Decree no. 250 of 2012, in the art. no. 13 ‘amendments to Annex VI of D.Lgs 155’ replaces the reference methods with those established by the European Standardization Committee (Republic of Italy 2012b). Finally, with Law No. 93 of 12 July 2022, Italy transposed the Stockholm Convention (Republic of Italy 2022).  The authors summarise in Table 11 the various pieces of legislation that have been enacted in Italy since 1966 on the subject of BD.

**Table 11:** List of Italian regulatory acts on BD

Regulatory act	Year	Title	References
Low no. 615	1966	Measures against air pollution	(Republic of Italy 1966)
Decree of the Ministry of the Environment and Health of 20 May 1991	1991	Criteria for the collection of air quality data	(Republic of Italy 1991)
D.Lgs no. 152/2007	2007	Implementation of Directive 2004/107/EC concerning arsenic, cadmium, mercury, nickel and polycyclic harmonic hydrocarbons in ambient air	(Republic of Italy 2007)
D.Lgs no. 155/2010	2010	Implementation of Directive 2008/50/EC relating to ambient air quality and cleaner air in Europe	(Republic of Italy 2010)
Ministerial Decree of 29 November 2012	2012	Identification of the special air quality measurement stations envisaged by article 6, paragraph 1, and article 8, paragraphs 6 and 7 of D.Lgs 155	(Republic of Italy 2012a)
D.Lgs no. 250/2012	2012	Amendments and additions to the Legislative Decree of 13 August 2010, n. 155	(Republic of Italy 2012b)

In Italy, multiple BD monitoring activities, summarised in Tables 12, 13, and 14, have been carried out over the past 20 years. Authorities, mainly the ISS, ARPAs, and Health Authorities, have carried out monitoring of BD and associated organic and inorganic micropollutants at various locations in Italy. These activities were carried out as part of environmental surveillance procedures, especially in industrial areas with different emission sources (incineration plants, thermoelectric power plants, steel mills, metallurgical industry). The Italian academic community has also carried out assessments of BD in various cities and industrial locations in recent years. Obviously, the micropollutants associated with the various industrial production cycles (PCDD/Fs, metals) and typical of urban environments (PAHs) have been focused on. In this section, the authors report the work carried out with the respective levels of micropollutants found in BD in different Italian areas.

**Table 12:** PCDD/F values in pg I-TE m^−2^ day^−1^ in BD in Italian locations (regions) with different anthropogenic impact. # in Fig. 2 indicates the location on the map of Italy

Locality (region)	# in Fig. 2	Area	PCDD/Fs	References
Aosta (Aosta Valley)	1	Industrial (steelplant)	3.7	(ARPAVDA 2008)
Aosta (Aosta Valley)	1	Urban	0.6–3.0	
Borgo Valsugana (Trento)	3	Industrial (steelplant)	0.57–4.07	(Rada et al. 2014)
Coriano (Emilia-Romagna)	6	Industrial (incinerator)	2.5	(Vassura et al. 2011)
Coriano (Emilia-Romagna)	6	Rural	1.2	
La Spezia (Liguria)	5	Industrial	0.09–0.41	(ARPAL 2023)
La Spezia (Liguria)	5	Urban	0.02–0.06	
Mantua (Lombardy)	2	Industrial	1.2–5.1	(Viviano et al. 2006)
Mantua (Lombardy)	2	Background	1.3–2.7	
San Nicola di Melfi (Basilicata)	13	Industrial	1.7–2.1	(Bove et al. 2005)
San Nicola di Melfi (Basilicata)	13	Rural	1.2–2.7	
Civitavecchia (Latium)	10	Industrial (harbour, LCP**)	0.05–0.14	(Settimo et al. 2021)
Civitavecchia (Latium)	10	Urban	0.05–0.46	
Civitavecchia (Latium)	10	Rural	0.07–0.20	
Perugia (Umbria)	8	Urban	1	(ARPAU 2023a)
Taranto (Apulia)	14	Industrial (steelplant)	12.9	(ARPAP 2019)
Taranto (Apulia)	14	Urban	4.8	
Taranto (Apulia)	14	Background	2.9	
Terni (Umbria)	9	Industrial (steelplant)	0.5–4.0	(ARPAU 2019)
Terni (Umbria)	9	Urban	1	(ARPAU 2023a)
Terni (Umbria)	9	Industrial (steelplant)	1.5	
Venice (Veneto)	4	Industrial	0.1–5.2*	(Rossini et al. 2005)
Venice (Veneto)	4	Urban	0.2–9.2*	
Venice (Veneto)	4	Lagoon	0.1–4.7*	
Viggiano (Basilicata)	15	Industrial (petrolchemical)	10.3	(ARPAB 2021)

^*^Values are calculated using WHO-TEF_1998_

**large combustion plant

**Table 13:** BaP values in ng m^−2^ day^−1^ in BD in Italian locations (regions) with different anthropogenic impact. # in Fig. 2 indicates the location on the map of Italy

Locality	# in Fig. 2	Area	BaP	References
Borgo Valsugana (Trento)	3	Urban	1–32	(Argiriadis et al. 2014)
La Spezia (Liguria)	5	Industrial	0.8–1.0	(ARPAL 2023)
La Spezia (Liguria)	5	Urban	0.8–1.0	
Naples (Campania)	12	Urban	1.2	(Qu et al. 2019)
Civitavecchia (Latium)	10	Industrial (harbour, LCP*)	1.4–4.6	(Settimo et al. 2021)
Civitavecchia (Latium)	10	Urban	1.4–7.1	
Civitavecchia (Latium)	10	Rural	1.4–3.9	
Rome (Latium)	11	Urban	1.6	(Ianiri et al. 2023)
San Nicola di Melfi (Basilicata)	13	Industrial	4.6–6.9	(Menichini et al. 2006b)
San Nicola di Melfi (Basilicata)	13	Urban	3.2–4.1	
San Nicola di Melfi (Basilicata)	13	Background	1.9–5.7	
Taranto (Apulia)	14	Industrial (steelplant)	79.4–135.6	(ARPAP 2019)
Taranto (Apulia)	14	Urban	58.5–145.7	
Taranto (Apulia)	14	Background	5.2–33.3	
Terni (Umbria)	9	Urban	10	(ARPAU 2023b)
Terni (Umbria)	9	Industrial (steelplant)	17	
Venice (Veneto)	4	Urban	30	(Rossini et al. 2005)
Venice (Veneto)	4	Rural	6–9	
Viggiano (Basilicata)	15	Industrial (petrolchemical)	1.3–4.5	(ARPAB 2021)

^*^Large combustion plant

**Table 14:** Metal values in µg m^−2^ day^−1^ in BD in Italian locations (regions) with different anthropogenic impact. # in Fig. 2 indicates the location on the map of Italy

Locality	# in Fig. 2	Area	As	Cd	Hg	Ni	Pb	Tl	Zn	V	References
Aosta (Aosta Valley)	1	Industrial (steelplant)	1.20	0.25	-	110	-	-	-	-	(ARPAVDA 2021)
Aosta (Aosta Valley)	1	Urban	0.70	0.10	-	10	-	-	-	-	
Augusta (Sicily)	17	Industrial	0.14	0.02	-	0.56	0.16	0.02	15.2	-	(Brugnone et al. 2023)
Gubbio (Umbria)	7	Industrial	0.4	0.2	-	3.4	3.7	-	-	-	(ARPAU 2023c)
La Spezia (Liguria)	5	Industrial	0.56	0.48	0.060	11	12	0.03	-	-	(ARPAL 2019)
La Spezia (Liguria)	5	Industrial	0.43–1.70	0.01–0.14	0.001–0.025	12.9–21.0	9.6–19.8	0.008–0.048	-	7.7–21.0	(ARPAL 2023)
La Spezia (Liguria)	5	Urban	0.13–1.23	0.01–0.07	0.001–0.010	0.8–6.8	1.4–12.6	0.001–0.037	-	0.8–13.7	
Mantua (Lombardy)	2	Industrial	-	0.2–0.4	0.24–0.55	3.1–7.6	4.7–14.0	-	-	-	(Viviano et al. 2006)
Milazzo (Sicily)	16	Industrial	0.26	0.04	-	1.08	0.66	0.04	32.6	-	(Brugnone et al. 2023)
Milazzo (Sicily)	16	Urban	0.15	0.03	-	0.36	0.48	0.03	23.7	-	
Perugia (Umbria)	8	Urban	0.20	0.60	-	3.8	4.3	-	152	-	(ARPAU 2019)
Perugia (Umbria)	8	Urban	0.30	0.60	-	2.0	3.9	-	-	-	(ARPAU 2023c)
Siracusa (Sicily)	18	Urban	0.17	0.03	-	0.49	0.14	0.02	16.9	-	(Brugnone et al. 2023)
Taranto (Apulia)	14	Industrial (steelplant)	3.0	0.80	-	13.6	93.0	1.01	394	-	(ARPAP 2019)
Taranto (Apulia)	14	Urban	1.30	0.30	-	5.5	28.1	0.26	220	-	
Taranto (Apulia)	14	Background	0.45	0.29	-	1.8	3.3	0.31	28	-	
Terni (Umbria)	9	Industrial (steelplant)	0.70	0.60	-	88.4	26.4	-	251	-	(ARPAU 2019)
Terni (Umbria)	9	Urban	0.40	0.50	-	27.8	7.5	-	132	-	
Terni (Umbria)	9	Industrial (steelplant)	1.5	0.3	-	79.2	38.7	-	-	-	(ARPAU 2023c)
Terni (Umbria)	9	Urban	0.5	0.3	-	17.6	7.0	-	-	-	
Venice (Veneto)	4	Industrial	0.82	0.34	-	6.9	7.6	-	97	-	(Rossini et al. 2005)
Venice (Veneto)	4	Urban	1.33	0.68	-	6.4	14.2	-	98	-	

Table 12 shows that PCDD/Fs values found in industrial areas are not always higher than in urban and rural areas. This may be explained by the fact that, especially during the winter period, both domestic heating systems and biomass combustion take place in such areas, together with the constant movement of internal combustion vehicles. Some work in the literature specifies that it is possible to have higher PCDD/Fs values in BD in rural sites than in industrial sites (Cappelletti et al. 2016; Wang et al. 2023). The Italian Institute for Environmental Protection and Research (ISPRA) specifies in a 2019 report that the largest source of dioxin and furan emissions are ‘non-industrial combustions’, understood as combustion processes aimed at producing heat for non-industrial activities (e.g. biomass combustion processes in chimneys and stoves, agricultural practices and uncontrolled combustion of waste in the air) (ISPRA 2019). These processes occur in most cases in urban and rural areas, contributing to the emission and subsequent release of PCDD/Fs into the environment through BD and passage into the food chain (Krause et al. 2022). As for PCDD/Fs, the authors report in Table 13 the values of BaP in BD found in various Italian areas.

Firstly, it should be noted that papers dealing with the PAH determination in BD are relatively scarce. Looking at the data, the amounts of BaP in BD tend to be higher in industrial areas than in urban and rural areas. The amounts of BaP and total PAHs in BD appear to be influenced by the season in which the monitoring is carried out. Several works show that the amounts of BaP in BD are higher during the winter season due to weather conditions (low temperatures and reduced solar window) and the greater presence of combustion sources (domestic heating systems, stoves, and chimneys) (Menichini et al. 2007; Settimo et al. 2021). BaP values in BD in Italian locations are in line with values found in other European areas. Moreover, taking the maximum permitted annual limit for total BaP deposition in Germany (500 ng m^**−**2^ day^**−**1^) as a legislative reference, all the values reported turn out to be lower than the latter. In conclusion, the authors report in Table 14, the values of metals found in BD among different areas of Italy.

**Fig. 2:**
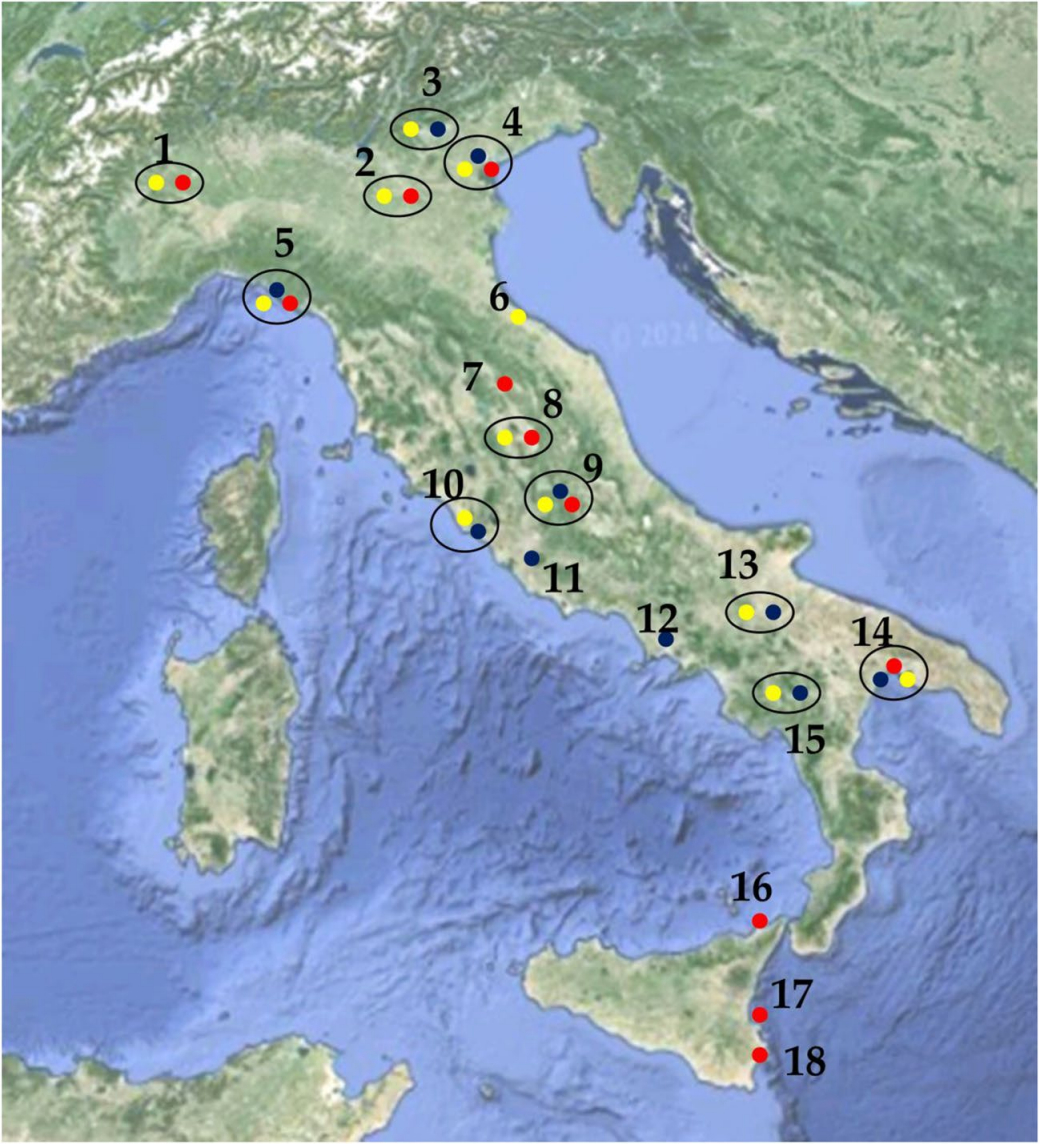
Locations where DB monitoring activities were conducted. 1: Aosta, 2: Mantua, 3: Borgo Valsugana, 4: Venice, 5: La Spezia, 6: Coriano, 7: Gubbio, 8: Perugia, 9: Terni, 10: Civitavecchia, 11: Rome, 12: Naples, 13: San Nicola di Melfi, 14: Taranto, 15: Viggiano, 16: Milazzo, 17: Augusta, and 18: Siracusa. Yellow, blue, and red: monitoring the BD of PCCD/Fs, BaP, and metals, respectively

The inorganic component (metals) in BD also tends to have higher concentrations in industrial areas than in urban and rural areas. The values in Table 14 show that the concentrations of As, Cd, Hg, Pb, Tl, and Zn found in Italian locations are below the current legal limits set by other European countries. For Ni, on the other hand, values exceeding the limits set by Germany and Croatia by 15 µg m^**−**2^ day^**−**1^ were often recorded. The values of metals, including As, Cd, and Ni, covered by Directive 2004/107/EC, determined in monitoring activities in various Italian areas (industrial, urban, and rural) are in line with data produced in other European countries. The authors in Fig. 2 displayed on the map of Italy the cities where the monitoring reported in this paper was conducted.

As can be seen, the BD monitoring and study activities conducted in Italy have been numerous despite the absence of specific limits. In any case, it should be considered that the adoption of specific limit values for BD may be essential to limit the intake of organic and inorganic micropollutants through the diet and consequently protect the health of the population.

## Conclusions

Determination of deposition fluxes is an essential tool for assessing population exposure to organic and inorganic micropollutants through the diet and consequently estimating potential health impacts. The monitoring of BD is even more important in areas where there are numerous emission sources such as industrial sites and large urban centres where pollutants, in addition to being suspended (adsorbed on SPM or in the gaseous phase), can be deposited on the ground and soil. Awareness that BD can be a health problem has prompted International and European authorities to develop both programmes (CLRTAP, OSPAR, UNEP, Stockholm Convention) and legislation (Directive 2004/107/CE and 2008/50) for increasing the knowledge of the deposition role on environment and population health. Despite numerous research BD programmes, at European level, there is still no a unique regulation establishing limit values or requiring member states to adopt limits to be used in environmental monitoring and risk assessment activities. Therefore, the present contribution aims to provide an overview of the BD legislative references already present in some European countries in order to highlight the need to adopt a sole legislation taking into account the latest WHO recommendations. Recently, the EU, through its proposal for a new directive on ambient air quality (Parliament and European Council 2022; European Council 2024), specifies that member states must periodically provide the Commission with new data on pollutant deposition by setting up monitoring stations called ‘supersites’ on their territory. The aim of such activities is that in the next few years, through a legislative framework dedicated to BD, the ground deposition fluxes of POPs could be reduced: consequently, an intake decrease of such substances should be achieved, safeguarding the human health. Some European countries, including Germany, Belgium, Croatia, Austria, and Slovenia, have in recent years established limit values for BD particulate matter (PM) and associated micropollutants (PCDD/Fs, DL-PCBs, BaP and metals). These values were adopted from monitoring data obtained over the years and taking into account territorial specificities (e.g. metoclimatic conditions). In Italy, no specific limit value for depositions has been adopted. In any case, some authorities such as ISS and ARPA have conducted multiple BD monitoring campaigns on Italian territory in recent years, producing data that can be considered as reference in environmental monitoring. According to the authors’ opinion, there is no sufficient attention to the BD topic. Data present in literature are not enough to draw a homogeneous picture of the situation, also due both to the sampling complexity and to the limited availability of adequate analytical instrumentation. In fact, the analytical determinations of POPs and metals in BDs are complex, time-consuming, require qualified personnel, and dedicated instruments. In order to provide sensitive and reliable results, the analysis must be carried out in strict accordance with the procedures reported in the different CEN standards. It often happens that some sampling and analysis operations are not conducted correctly. The lack of BD data concerns not only Italy but generally all EU countries. It is now 20 years since the introduction of the first piece of legislation on the subject of BD (Directive 2004/107/CE) and there is still no dedicated law. Through this work, the authors wish to urge, firstly, the scientific community to implement monitoring activities and data on BD and, secondly, the authorities to act accordingly through the creation of a dedicated law.

## Data Availability

The authors declare full availability to share data.
